# Using deep learning to identify maturity and 3D distance in pineapple fields

**DOI:** 10.1038/s41598-022-12096-6

**Published:** 2022-05-24

**Authors:** Chia-Ying Chang, Ching-Shan Kuan, Hsin-Yi Tseng, Pei-Hsuan Lee, Shang-Han Tsai, Shean-Jen Chen

**Affiliations:** 1 Bachelor of Program in Scientific Agriculture, National Pingtung University of Science and Technology, Pingtung, 91201 Taiwan; 2 Chiayi Agricultural Experiment Branch, Taiwan Agricultural Research Institute, Chiaya, 600015 Taiwan; 3 Plant Germplasm Division, Taiwan Agricultural Research Institute, Taichung, 413008 Taiwan; 4 General Research Service Center, National Pingtung University of Science and Technology, Pingtung, 91201 Taiwan; 5 College of Photonics, National Yang Ming Chiao Tung University, Tainan, 711 Taiwan

**Keywords:** Computational science, Computer science

## Abstract

Pineapples are an important agricultural economic crop in Taiwan. Considerable human resources are required to protect pineapples from excessive solar radiation, which could otherwise lead to overheating and subsequent deterioration. Note that simple covering all of the fruit with a paper bag is not a viable solution, due to the fact that it makes it impossible to determine whether the fruit is ripe. This paper proposes a system by which to automate the detection of ripe pineapples. The proposed deep learning architecture enables detection regardless of lighting conditions, achieving accuracy of more than 99.27% with error of less than 2% at distances of 300 ~ 800 mm. This proposed system using an Nvidia TX2 is capable of 15 frames per second, thereby making it possible to mount the device on machines that move at walking speed.

## Introduction

Pineapple have been grown commercially in Taiwan for over 300 years. It is a perennial herbaceous fruit well-suited to cultivation in the hilly areas of central and southern Taiwan; however, it is grown in flat areas for the purposes of mass cultivation. Taiwan has bred a number of pineapple varieties with different production periods. By 2002, Taiwan output had reached 2.56 million metric tons and by 2018, annual earnings had reached 43 million US dollars.

Pineapples are multi-flowered fruits, and although any given batch of pineapple fields may undergo flowering at roughly the same time, the times at which the florets opens can vary widely. Under these conditions, it is often necessary to harvest a single field three or four times. After flowering, pineapple^1^ enters the fruit development (BBCH code 7) period, during which the fruit begins to develop and expand. The fruit head gradually changes from conical to smooth, and the color of the peel gradually changes from reddish brown (BBCH code 703) to dark green (BBCH code 705). During the final stage of development (BBCH code 709), the size of the fruit does not change but the color of the peel changes from dark green to light green. In the fruit-ripening stage (BBCH code 8), the fruit gradually turns yellow and the emits the aroma of pineapple. Commercially-available pineapple fruit can be divided into solid-sounding fruit, hollow-sounding fruit and semi-hollow-sounding fruit^2^, in accordance with the sound that is reflected when the fruit is tapped. Solid sound fruit is characterized by low acidity, thick meat, and a bright golden color. The solid sound fruit was more mature than hollow sound, and the suitable harvest time was appeared light green sprinkled with white powder (when the peel turns yellow, it is too ripe). Hollow sounding is characterized by lower water content, thinner fibers, strong sweetness, string acidity, strong fragrance, and a light milky yellow. The suitable harvest time was ripening from green to yellow. The semi-hollow-sound is somewhere in between.

Mechanization has not been widely adopted for the harvesting of fruit from pineapple fields^3^. The lack of automated systems by which to assess the ripeness of fruit necessitates the presence of experienced professionals in the field during harvesting. Note that non-destructive sugar content detectors developed specifically for this purpose cannot replace professionals in the field. Sourcing workers for the harvesting of pineapple fruit is a perennial problem, due to the fact that the work requires heavy manual labor as well as experience in fruit identification.

It is not uncommon to cover ripening pineapples using paper hats, paper bag or blackening nets for sun protection and the prevention of dehiscence. Unfortunately, coving the fruit this way makes it impossible to determine the ripeness of the fruit without manually lifting the bag and checking with the naked eye^4^. The system developed in the current study was based on the assumption that the paper hats leave at least half of the fruit visible from beneath.

Numerous researchers have investigated methods by which to determine the maturity and varietal of pineapples in the field. Azman et al. used a convolutional neural network (CNN) for the indoor assessment of ripeness (unripe, partially ripe, and fully ripe), achieving classification accuracy of 94% when applied to 27 samples with known ripeness^5^. Angel et al. used color analysis and image recognition software to classify pineapple maturity into 11 levels when viewed under indoor conditions^6^. They achieved classification accuracy of 96% for 550 pineapple samples. Researchers in Thailand examined two pineapple varieties using gradient analysis of textures^7^. Sanyal et al. reported on the use of computer technology to diagnose different diseases aimed at increasing the production of pineapples^8^. Amjad et al. used VGG16 in identifying bromeliads, achieving classification accuracy of 100%^9^. Edwin et al. used image processing and fuzzy theory to classify post-harvest maturity into four categories. They achieved classification accuracy of 90% for partial maturity and 95% overall accuracy^10^. Note however that all of the methods described above rely on a controllable light source under indoor conditions. Wan et al. used a UAV equipped with a color camera to determine the number of pineapple crowns in a field. They achieved classification accuracy of 94%^11^.

Deep learning is a machine learning technique that uses multi-layer artificial neural networks to analyze signals. In the current study, we employed a CNN for the real-time detection of pineapple ripeness in the field as follows: unripe, ripe, ripening, and bagged. Unripe: un-harvestable, the fruit was not final the development and not to reaches the final size. Ripe: The solid sound fruit must be harvested when the dark green peel turns to light green, and the fruit has reached its final size at this time. Ripening: the peel turns to yellow. Bagged: the fruit can’t be saw the peel and can’t be identify the mature.

To deal with the difficulties of real-time computing and cost, we employed an embedded system NVIDIA TX2 in implementation of the YOLO^12^ pre-trail network. The Intel D435i^13^ camera was the input data to TX2, it has RGB data and depth data for each frame. We assume that this device (including cameras and embedded systems) was built under the platform of the elevated tracked vehicle and attached to a simple harvesting mechanism.

## Results

In this section, we discuss the effects of training on recognition performance for different databases (Table 1). We also discuss the means by which to assess accuracy in classification and identification tasks.

**Table 1:** Test results using various training databases.

Classes	Database 1				Database 2				Database 3			
	Train		Test		Train		Test		Train		Test	
	CA (%)	BA (%)	CA (%)	BA (%)	CA (%)	BA (%)	CA (%)	BA (%)	CA (%)	BA (%)	CA (%)	BA (%)
Bag									100	100		
Ripe					97.71	100	96.70	100	98.86	100	100	100
Ripening	100	99.75	95.24	100	100	99.75	95.24	100	99.75	100	100	100
Unripe	100	100	96.71	98.82	99.17	100	77.34	97.64	98.33	100	77.38	99.12

(CA: classification accuracy; BA: box selection accuracy).

### Deep learning

During training, root mean square error (RMSE) was used for evaluation, based on the standard deviation of the residual, where values that are closer to zero indicate superior accuracy.

#### Accumulative database

**Figure 1:**
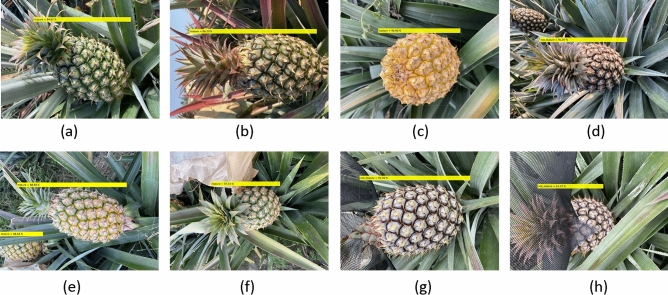
Experiment results: (**a**–**d**) training images; (**e**–**h**) test images.

We categorized the fruit as ripening or unripe using 518 training images and 360 testing images. The results were as follows: training time (19.2 h), loss (0.0042), and RMSE (0.06). The classification accuracy (CA) and box selection accuracy (BA) are shown in Fig. 1. In training database as Fig. 1a–d, a–c can be correctly classified as ripening and (d) as unripe, and the confidence score for each image is 94.61%, 97.79%, 96.96%, and 91.07%. In testing database as Fig. 1e–h, e,f can be correctly classified as ripening, and (g) and (h) are unripe, and the confidence score for each image is 88.68%, 86.20%, 95.93%, and 61.37%. The results in the testing dataset had lower confidence than those in the training dataset, such as (g) and (h) are the fruits sunhats without in the training set, but they can be correctly distinguished and positioned in both frame selection and classification. Especially in the Fig. 1c, the all-yellow fruit without crowns is mainly due to the rapid changes in temperature, resulting in the reduction or disappearance of the growth point differentiation ability, which was appear in the actual field. Our database was generated for on-site assessment. In the future, the database of fruit development that will appear in field-related field management will continue to increase. In Table 2, the first part (database 1), the classification accuracy (CA) and box selection accuracy (BA) of unripe fruits (120 images) were 100% in the training set. However, one of the ripening fruits (total 398 images) fails to correctly selection the box of the fruit, and the rest of the box selection fruits can be correctly selected. In the test set, the CA of unripe fruits (21 images) was 100%, and only one of them was misclassified. However, among the ripening fruits (339 images), 4 images of them failed to correctly selection the box of the fruit, so the BA was 98.82%, among the 335 correct box selections, 11 images were classified incorrectly, so the CA was 96.71%.

We also categorized the fruit as unripe, ripening, or ripe using 693 training images and 572 test images. The results were as follows: training time (25.6 h), loss (0.0036), RMSE (0.06). For the additional category (i.e., unripe), framing of the pineapple was successful (i.e., the pineapples were correctly positioned in the frame), but some of the ripe fruits were classified as unripe due to similarities in color. In Table 2 (database2), 4 images in the ripe training set (175 images) were misclassified as ripe, and the CA was 97.91%, and the ripening BA was 99.75% which the one image fail to detect the fruit in ripening set (398 images). In the training part of unripe, the BA was 100%, but CA was 99.17% which one image misclassified as ripe. Among the 339 images of unripe fruit in test set, pineapple detection failed in 8 images (BA was 97.64%), 74 were misclassified as ripe and five were misclassified as ripening (CA was 77.34%). In the ripening test set, 21 images can be detected the fruits, but one image fail to classified as unripe (CA: 95.24% and BA:100%). Among the 212 test images of ripe, seven were misclassified as unripe (CA:96.70% and BA:100%).

**Table 2:** 3D distance experiment: indoor. Significant values are in bold.

	Ruler (mm)	Laser rangefinder (mm)	Depth distance (mm)	Standard points		Sampling point		D_x_ (mm)	Error rate of D_x_	D_y_ (mm)	Error rate of D_y_	D_z_ (mm)	Error rate of D_z_	△D_z_(-6 mm)	Error rate of △D_z_
				X_1_	Y_1_	X_b_	Y_b_								
1	250	244	255	960	540	1298	201	56.10	**2.71**	− 59.85	**1.23**	+ 11	**4.51**	+ 5	**2.05**
2	300	295	302	951	543	661	259	− 57.00	**1.99**	− 59.38	**1.20**	+ 7	2.37	+ 1	0.34
3	350	344	352	985	548	1238	704	57.96	1.43	38.02	1.13	+ 8	2.33	+ 2	0.58
4	400	396	405	956	551	730	343	** − 59.58**	0.85	− 58.32	1.15	+ 9	2.27	+ 3	0.76
5	450	445	456	981	545	790	680	− 56.69	1.38	42.62	0.14	+ 11	**2.27**	+ 5	1.12
6	500	493	504	958	553	1124	374	54.46	1.70	** − 62.46**	**0.11**	+ 11	2.23	+ 5	1.01
7	550	545	552	947	554	793	666	− 55.33	1.39	42.80	0.15	+ 7	1.28	+ 1	0.18
8	600	593	605	960	548	1106	642	57.49	0.91	39.37	0.43	** + 12**	2.02	** + 6**	1.01
9	650	646	650	939	555	800	427	− 58.81	0.65	− 57.60	0.83	+ 4	0.62	** − 2**	0.31
10	700	693	699	961	555	833	436	− 58.24	0.68	− 57.59	0.77	+ 6	0.87	0	**0.00**
11	750	742	750	953	556	1072	628	**58.09**	0.65	37.39	0.62	+ 8	1.08	+ 2	0.27
12	800	791	795	949	520	1061	625	57.95	**0.63**	**57.79**	0.65	+ 4	**0.51**	** − 2**	0.25

We also categorized the fruit as unripe, ripening, ripe, or bagged using 928 training images and 572 test images. The results were as follows: training time (34.5 h), loss (0.0056), RMSE (0.07). The position of the fruit was correctly framed in the training image set. Training images: 1) In classifying ripe pineapples, one image was misclassified as unripe and one image was misclassified as ripening (CA:98.86%). 2) In classifying ripening pineapples, one image was misclassified as bagged (CA:99.75%). 3) In classifying unripe pineapples, two images were misclassified as ripe (CA:98.33%). The position of the fruit was correctly framed in the test image. In classifying ripe and ripening pineapples, all images were correct. In unripe 339 images, 3 images of them failed to correctly selection the box of the fruit, so the BA was 99.12%. In classifying the 339 unripe images, one image was misclassified as ripening, one image was misclassified as bagged, and 75 images were misclassified as ripe (CA: 773.8%).

Increasing the number of images in the database was shown to improve detection performance. Our inability to exceed 95% accuracy can be attributed to the fact that ripe pineapples appear similar to unripe pineapples.

#### 3D Distance

We performed experiments under indoor and outdoor testing environments. In the indoor test, 3D distance estimates were verified using a checkerboard grid with cells measuring 21 mm. The accuracy of 3D distance estimates could not be obtained under outdoor conditions; therefore, we used only D_z_ distance error.

**Figure 2:**
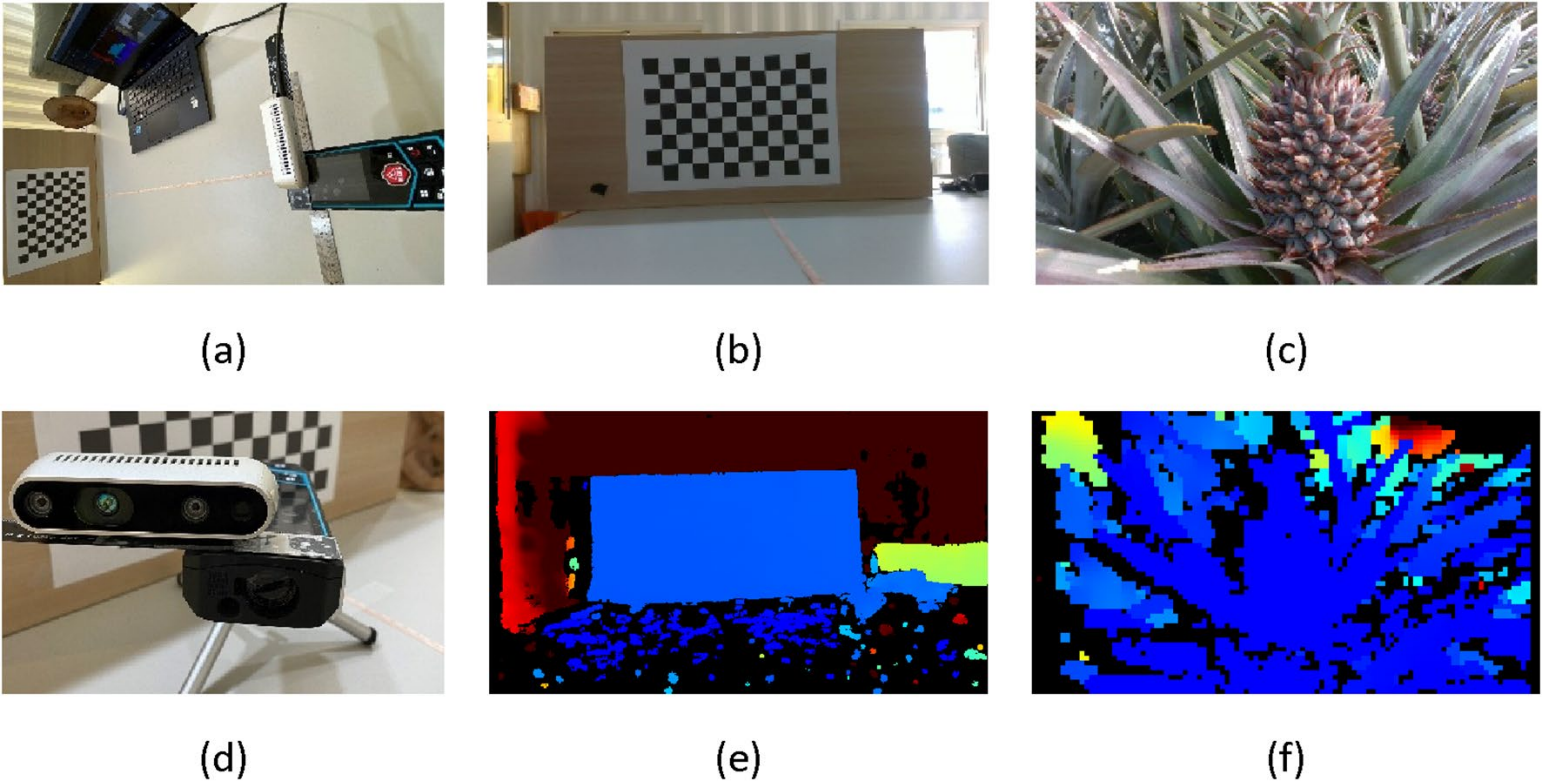
Distance experiment setup: (**a**) and (**d**) device architecture; (**b**) and (**e**)indoor experiment; (**c**) and (**f**) field experiment.

The checkerboard in the indoor test was maintained in a fixed position using a ruler and a laser rangefinder. Depth values were obtained using the depth camera (D434i). In order to verify the accuracy of the distance we calculated, we added the laser rangefinder as the standard answer, calculated the difference between the two as the calculation error (*D*), and divided the difference by the standard distance to obtain the error rate (error rate of D). Laser rangefinder The architecture and arrangement of the camera are shown in Fig. 2a and d.The experiment setup is shown in Fig. 2b and a depth map is shown in Fig. 2e. We used the intersection of black and white cells closest to the center of the screen as a center point for calculating the distance based on sampled coordinate points, the results of which are shown in Table 3. In the experiment, the Z-axis distance was 250 ~ 800 mm, D_x_ distance was + 58.09 mm and -59.58 mm, D_y_ distance was -62.48 mm (upper area) and + 57.79 mm (lower area), and D_z_ distance (laser range- depth camera) finder was + 12 mm. We can find that in the value of the Z axis, our calculation results are more than 4 mm larger than the distance of the laser rangefinder. Therefore, when we subtract 6 mm from the calculated values as △D_z_, the error rate of △D_z_ are all less than 1.12% at the distance of 300 ~ 800 mm. As shown in Table 3, over a distance range of 300 ~ 800 mm, 3D distance error was less than 2%, which means that under Z-axis distance of 300 mm, the maximum error would be 6 mm.

**Table 3:** Z-axis distance experiment: outdoors. Significant values are in bold.

	Laser distance (mm)	Depth distance (mm)	Difference (mm)	Error rate (%)		Laser distance (mm)	Depth distance (mm)	Difference (mm)	Error rate (%)
1	196	199	3	**1.53**	6	391	385	6	**1.53**
2	287	291	4	1.39	7	466	467	1	0.21
3	307	306	1	0.33	8	554	558	4	0.72
4	311	311	0	**0.00**	9	684	689	5	0.73
5	315	316	1	0.32					

In the outdoor experiment, we attached the laser rangefinder aligned with the x-axis of the camera, as shown in Fig. 2c and f. During this test, we were unable to obtain precise distance measurements along the x- and y-axes; therefore, we tested accuracy only along the z-axis. Note that the position of the pineapple does not necessarily match the point measured by the laser rangefinder and the body of the pineapple is arc-shaped. We placed Fig. 2d in a pineapple field for testing, placed the camera at nine different distances and positions, and tested the difference from the laser rangefinder and our system. Thus, the maximum error obtained from nine groups of test results was 1.53% (see Table 1).

The YOLO V2^12^ architecture made it possible to detect pineapple maturity in 99.27% of cases. In 3D distance experiments, error in 3D distance estimates was less than 2% at 300 ~ 800 mm in an indoor environment. Z-axis distance estimates obtained in the field were consistent with our indoor test results, resulting in error of 1.53%.

## Discussion

This paper presents preliminary work on the development of an automated system for the harvesting of pineapples. We used a database of images collected in the field (Tainung No. 17) for offline training. The detected fruits were classified as harvestable (ripe and ripening) or un-harvestable (unripe). Note that these assessments were obtained for fruits that were partially shaded from the sun using a paper bag. Performance in box selection reached 99.27% with 3D distance error of less than 2%. A failure to exceed classification accuracy of 95% prompted us to subdivide the ripe samples into the following two groups with the aim of enhancing classification accuracy: stage 709 and stage 8. Trained network weight files were transferred from an i7-1165G7 2.8 GHz host computer with no graphics card to an embedded system equipped with an NVIDIA TX2, thereby increasing the frame rate from 5 to 15 FPS (including distance conversion). The distance data was then transferred to the harvesting machine via an RS232 transmitter.

**Figure 3:**
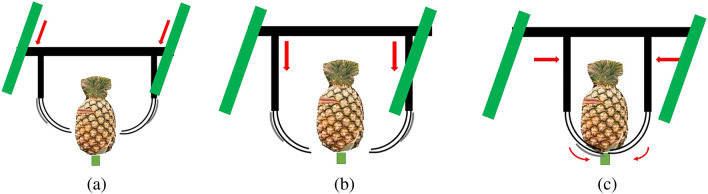
Preliminary plan for simple harvesting mechanism: (**a**) lateral alignment of harvesting mechanism; (**b**) lowering of clamping device; (**c**) closure of clamping device around stem beneath fruit.

We expect to improve on the poor recognition rate achieved in the test set in the future. We will also augment the database by adding more data pertaining to inflorescence, fruit development period, and pineapple varieties. The preliminary design and planning of the simple harvesting machinery is shown in Fig. 3. The harvester moves back and forth to align the attached slides (green in the image) with the fruit, as shown in Fig. 3a. After alignment, the two semi-circular halves of the harvester device are then lowered to surround the fruit, as shown in Fig. 3b. The two halves are then moved to within a set distance of the stem, whereupon an electric cutting device picks the fruit, as shown in Fig. 3c.

## Methods

Offline training was conducted using Matlab 2021b running on a PC equipped with an Intel i9-11900F and RTX-3080Ti graphics card with the weight files held in an embedded NVIDIA TX2. In the following, we describe how we obtained the database and then outline the network architecture and our reasons behind its selection. Finally, we describe the means by which detection data was converted into 3D distances.

### DataBase

The most important part of any deep learning implementation is the collection of data and the accuracy of ground truth values. Collecting data in the field can be time-consuming; therefore, we discussed the issue with Ching-Shan Kuan and Hsin-Yi Tseng at the Taiwan Agricultural Research Institute before initiating the collection process. Data collected in the field were classified and labelled in accordance with BBCH^1^ codes throughout the growing period. The correctness of the data was confirmed manually. We compiled a database of 8,852 images, as follows: stage 5 (2186), stage 6 (2176), stage 7 (3,449), stage 701 (1990), stage 705 (1002), stage 709 (457), and stage 8—ripening (419), and stage 8—ripe (387). We also collected 235 images of bagged pineapples. The database data we used was shown in Table 4. It was classified according to the BBCH, and randomly divided into training set and test set. Table 4 lists the categories and the number of images in each. Our main objective was to assess fruits in the fields during the final stage.

**Table 4:** Database categories and number of images based on BBCH code. Significant values are in bold.

BBCH Code	Description	AI Labels	Number of images	Database 1		Database 2		Database 3	
				Train	Test	Train	Test	Train	Test
Not in category	Bagged to prevent excessive sun exposure	**Bag**	235					235	0
800	Ripe: still green	Ripe (**green**)	387			175	212	175	212
801 ~ 809	Ripening: gradually yellowing	Ripening (**mature**)	419	398	21	398	21	398	21
709	Unripe: immature	Unripe (**not mature**)	459	120	339	120	339	120	339
Total images			**1500**	518	360	693	572	928	572

**Figure 4:**
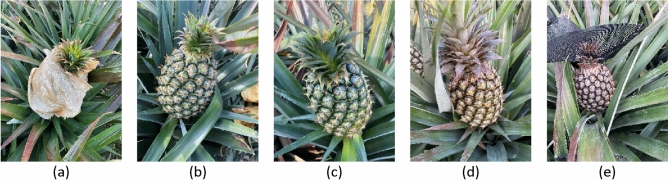
Database categories: (**a**) bag; (**b**) ripe; (**c**) ripening; (**d**) unripe; (**e**) sunhat.

The fruit was divided into four categories: bagged (Fig. 4a), ripe (Fig. 4b), ripening (Fig. 4c), and unripe (Fig. 4d). ‘Ripe’ refers to fleshy fruit that is entirely green but must be harvested. ‘Unripe’ refers to fleshy fruit and hollow-sounding fruit that are not harvestable. ‘Ripening’ refers to hollow-sounding fruit undergoing a color change from green to yellow. The extent of color change can be subdivided according to maturity level. In Fig. 4b,c, the head of the fruit is slightly different, the head of (c) is slightly yellow, and turns from bottom to top; (b) is the whole fruit entirely green without turn yellow. Note that when paper bags are used to protect the fruit from sun exposure, it is not possible to determine the degree of maturity. Note that images showing sun hats (Fig. 4e) were not included in the training or test sets.

### Deep learning

The YOLO architecture is available in three versions: First generation^14^ (V1) unifies the two-stage solution (Class, Bounding Box) under the CNN architecture into a regression problem with calculation speed of 45 FPS; second generation^12^ (V2) offers improved accuracy, convergence, and calculation speed of 67 FPS; and third generation^15^ (V3) exhibits improved detection of small objects but is slower. The fourth generation^16^ (V4) was meant to find an optimal balance of resolution and the number of convolution layers. This enables a high recognition rate with high computing efficiency and speed of 65 FPS. V5^17^ is largely the same as V4; however, the computing speed is twice as fast.

Note that in this study, we performed detection using a single station in the orchard. The size of objects detected in the images exceeded the 300*300 px resolution of the camera and no more than two objects were ever included in the same frame. Thus, there was no need to detect multiple small objects. The background in the pineapple field was less complex and less variable than that in the COCO database. Thus, in the selection of a network architecture, our primary consideration was speed and accuracy. We opted for the YOLO V2 network architecture using models pre-trained in MATLAB. The parameter settings were as follows: output images (448*448*3) with anchor boxes (344,338), (108,115), (223,290), (238,167). The Yolonet parameters in experiments were as follows: feature layer (leakyrelu_24), mini-batch-size (32), max-epochs (200), learning rate (0.001). In order to avoid the overfitting problem, we randomly selected the training set in the database, and used the test images that are not in the training set to confirm whether there is overfitting.

### 3D Distance

**Figure 5:**
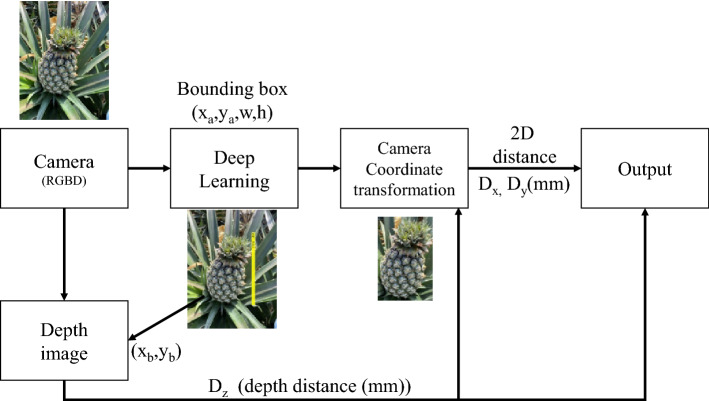
System flowchart.

Note that the selected camera uses left to right dual-camera parallax to estimate distances in conjunction with TOF technology to correct for distance error, resulting in Z-axis distance error of less than 2%^13^. Note also that the working range of the camera was from 20 cm to 10 m, which was in line with our needs. As shown in Fig. 5, after the camera recognizes the frame selection position of the object through deep learning, the center point of the frame is calculated to read the Z-axis distance of the corresponding pixel position. Convert the 3-axis actual distance (mm) from the obtained Z-axis distance and the screen frame selection center point.

After determining the position of the pineapple via deep learning, as shown in Fig. 5, we used the coordinates of the upper left corner of the frame (x_a_, y_a_) to calculate the center point position (x_b_, y_b_) as showed in Eq. (1). The distance value of the depth image was read from this position. To account for the risk of distance failure under strong lighting conditions, we sought to strengthen the depth values using Eq. (2). Thus, we used the eight adjacent points and the center point to calculate depth value $D_{Z}$. Once the camera is installed beneath the field harvester, the body of the machine should block strong light.

1 $$\left( {{\text{x}}_{b} ,y_{b} } \right) = \left( {{\text{x}}_{a} + \frac{w}{2},y_{a} + \frac{h}{2}} \right)$$

2 $$\begin{aligned} D_{Z} &= ({\text{depth image}}\left( {{\text{x}}_{b} - 1,y_{b} - 1} \right) + {\text{depth image}}\left( {{\text{x}}_{b} ,y_{b} - 1} \right) \\ &\quad + {\text{depth image}}\left( {{\text{x}}_{b} + 1,y_{b} - 1} \right) + {\text{depth image}}\left( {{\text{x}}_{b} - 1,y_{b} } \right) \\ &\quad + {\text{depth image}}\left( {{\text{x}}_{b} ,y_{b} } \right) + {\text{depth image}}\left( {{\text{x}}_{b} + 1,y_{b} } \right) \\&\quad + {\text{depth image}}\left( {{\text{x}}_{b} - 1,y_{b} + 1} \right) + {\text{depth image}}\left( {{\text{x}}_{b} ,y_{b} + 1} \right) \\ &\quad + {\text{depth image}}\left( {{\text{x}}_{b} + 1,y_{b} + 1} \right))/{\text{num }} \\ \end{aligned}$$

3 $${\text{num}} = {\text{Calculate the number of }}9{\text{ depth image values in }}D_{Z} {\text{ that is greater than }}1$$

D_z_ values were used to calculate the x-axis distance D_x_ and y-axis distance D_y_. Using the known camera FOV (H: 64°, V: 41°) and camera resolution (1920*1080 pixels), distance can be calculated using trigonometric functions via Eqs. (4–5). In the center of the screen, Dx = Dy = 0 mm, in the upper right corner of the screen, Dx > 0, Dy < 0, and in the lower left corner of the screen, Dx < 0, Dy > 0.

4 $$D_{x} = \frac{{\tan \left( \frac{H}{2} \right) \times dz \times 2}}{m} \times \left( {x_{b} - x_{1} } \right)$$

5 $$D_{y} = \frac{{\tan \left( \frac{V}{2} \right) \times dz \times 2}}{n} \times \left( {y_{b} - y_{1} } \right)$$

where (× 1,y1) indicate center point of the image, the first value was m/2, and the second value was n/2. The position of the object determined using deep learning can be converted into 3D distance (mm) using Eqs. (1–5).

## Data Availability

The database was collected by Dr. Ching-Shan Kuan and Dr. Hsin-Yi Tseng from the chiayi agricultural experiment branch and assistant researcher, plant germplasm division, Taiwan Agricultural Research Institute. Since the data is still being collected and the plan is being implemented, the information cannot be made public. If necessary, the network structure and program can be sent to the corresponding author, and the corresponding author will execute the feedback data.
